# A Comparative Investigation of Chemical Decontamination Methods for In-Situ Cleaning of Dental Implant Surfaces

**DOI:** 10.3390/jfb14080394

**Published:** 2023-07-25

**Authors:** Badra Hussain, Sadia Khan, Anne Eriksson Agger, Jan Eirik Ellingsen, Ståle Petter Lyngstadaas, Jaime Bueno, Håvard J. Haugen

**Affiliations:** 1 Department of Biomaterials, Institute of Clinical Dentistry, University of Oslo, 0317 Oslo, Norway; badra.hussain@odont.uio.no (B.H.); a.e.agger@odont.uio.no (A.E.A.); spl@odont.uio.no (S.P.L.); 2 Department of Prosthetics and Oral Function, Institute of Clinical Dentistry, University of Oslo, 0317 Oslo, Norway; s.n.khan@odont.uio.no (S.K.); janee@odont.uio.no (J.E.E.); 3 Section of the Postgraduate Program in Periodontology, Faculty of Dentistry, Complutense University of Madrid (UCM), 28040 Madrid, Spain; jaimebue@ucm.es

**Keywords:** dental implant, acquired pellicle, peri-implant disease, chemical decontamination

## Abstract

Surface chemistry evaluation is crucial in assessing the efficacy of chemical decontamination products for titanium implants. This study aimed to investigate the effectiveness of chemical decontamination solutions in cleaning a contaminated dental implant surface and to evaluate the potential of combining Pluronic gel with hydrogen peroxide (NuBone^®^Clean) by evaluating pellicle disruption and re-formation on implant surfaces. In addition, ensuring safety with in vitro and human testing protocols. X-ray Photoelectron Spectroscopy (XPS) was utilised for surface analysis. All the tested gels had some effect on the surface cleanness except for PrefGel^®^. Among the tested chemical decontamination candidates, NuBone^®^Clean demonstrated effectiveness in providing a cleaner titanium surface. Furthermore, none of the tested chemical agents exhibited cytotoxic effects, and the safety assessment showed no adverse events. The results of this study highlight the significance of conducting comprehensive evaluations, encompassing safety and efficacy, before introducing new chemical agents for dental treatments. The findings suggest that NuBone^®^Clean shows potential as a chemical decontamination solution for implant surfaces. However, further investigation through randomised clinical trials is necessary. By adhering to rigorous testing protocols, the development of safe and efficient chemical decontamination strategies can be advanced, benefiting patients and promoting progress in implant dentistry.

## 1. Introduction

Infections associated with implants represent a worldwide health concern [1]. With the ongoing demographic shift toward an ageing population [2], the prevalence of devices such as dental, hip, and knee prostheses, joint replacements, and screws used to substitute or reinforce deteriorating bone structures is increasing. A significant challenge associated with these applications is bacterial colonisation, either during the surgical procedure or after being in function. 

The surgical implantation exposes these devices to potential bacterial colonisation, originating from the patient’s skin, healthcare staff, or surgical tools [3]. Once the bacteria adhere to the implant’s surface, they can establish a stronghold over time, producing a viscous layer known as a biofilm [4]. This self-secreted barrier protects against the host’s immune response and antibiotic treatments, contributing to the rise in antibiotic resistance [5]. In cases where the body’s immune system or antibiotic treatment fails to eradicate the infection, further corrective surgeries are necessary [6], including a cleaning process of the implant. In some cases removing the implant is imperative [7]. 

An illustration of bacterial colonisation occurring after the implant has been in use is evident in peri-implant diseases, which affect dental implants [8]. These are biofilm-driven diseases involving multiple species of microorganisms [9]. These diseases are considered multifactorial, where the host response also plays an important role [10]. The initial step in biofilm formation on a dental implant surface is establishing a pellicle layer, providing a foundation for bacterial attachment and subsequent biofilm development [11]. Within seconds after cleaning the implant components above the bone level, an acquired pellicle forms on the exposed implant surface in the oral cavity. Bacterial adhesion and biofilm development depend on the composition and adhesion strength of the proteins adhering to the surface [12]. These proteins exhibit diversity in composition and adhesion strength, which can be influenced by numerous factors, including the surface chemistry of the material [13]. 

Managing peri-implant diseases poses significant challenges due to the lack of remedies specifically designed for treating the dental implant surface [14]. Titanium is the leading material for dental implants [15]; any treatment of this surface will affect the state of the surface on a macroscopic, microscopic and chemical level. This study aimed to evaluate various chemical decontamination solutions, with an evaluation of a detailed chemical characterisation of the titanium surface after using the cleaning agents. One of the remedies in this study, consisting of Pluronic and H_2_O_2,_ has not yet been clinically used. This study includes the assessment of the safety of this remedy through in vitro cell testing and clinical assessment. This comprehensive evaluation will allow us to understand better the cleaning agent’s effects on the titanium surface and potentially pave the way for more effective treatments for peri-implantitis.

## 2. Materials and Methods

### 2.1. Preparation of Titanium Discs and Decontamination Groups

Commercially pure titanium discs, measuring 6.2 mm in diameter and 2 mm in height, were subjected to grit-blasting and acid etching in a hydrofluoric acid solution (0.2 vol.%) to replicate the rough surface characteristics of a dental implant surface, precisely resembling the OsseoSpeed^®^ surface commercially available from Dentsply Sirona (Charlotte, NC, USA). The surface treatment protocol followed established procedures described by Lamolle et al. [16]. To ensure uniformity, all surfaces were analysed with a PLμ NEOX (Sensofar-Tech S.L., Terrassa, Spain) blue light laser profilometer and interferometer using a 50 × EPI (Nikon, Tokyo, Japan) confocal objective was used for assessing an extended topography of 2 × 2 images. Each had a viewing area of 253 μm × 190 μm at 20% overlapping. Eight images of each material were analysed. The advanced topography software Sensomap 5.1 Plus (Sensofar-Tech S.L., Terrassa, Spain) for dimensional and surface state metrology was used to process the measured data. The following surface amplitude parameters were analysed to ensure homogenous surfaces: average roughness (Sa), the total height of the surface (St), skewness of the height distribution (Ssk), kurtosis of the height distribution (Sku) and the maximum height of summits (Sp).

Six decontamination groups were evaluated (Table 1): PrefGel^®^ (Institut Straumann AG, Basel, Switzerland), Perisolv^®^ (Regedent AG, Zurich, Switzerland, 3% vol H_2_O_2_ (Sigma-Aldrich, Oslo, Norway), Pluronic gel (Sigma-Aldrich, Oslo, Norway), NuBone^®^Clean, (Corticalis AS, Oslo, Norway), and GUM^®^ Paroex^®^ (0.12% Chlorhexidine gluconate and 0.05% Cetylpyridinium chloride, Sunstar Suisse, Etoy, Switzerland).

### 2.2. Dental Pellicle Model—Pellicle Formation and Decontamination

**Figure 1:**
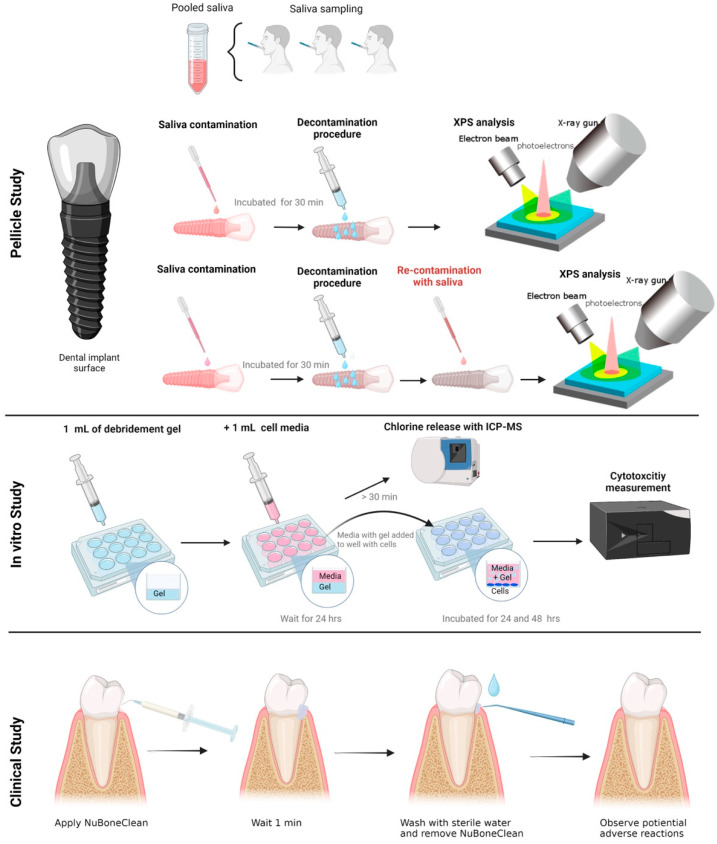
Graphical illustration of how the pellicle study (**top** panel), in vitro (**middle**) and clinical study (**lower** panel) was performed.

Saliva samples were collected from three healthy individuals, pooled together, and subjected to centrifugation. The prepared titanium discs were placed in 24-well plates (ThermoFisher, Waltham, MA, USA), and the pooled saliva was applied onto the disc surfaces. Following application, the discs were incubated at 37 °C for 30 min to facilitate the formation of a pellicle layer on the surface. It is important to note that the pellicle was not sterilised, although the presence of bacteria within it was not assessed in this study. To ensure the robustness of the experimental design, three discs were contaminated at three different time points for each decontamination group (n = 9). Two sets of discs were utilised for each sample in this study phase. One set was decontaminated and analysed, while the other set was first decontaminated and then re-contaminated with the pooled saliva before analysis after re-contamination (Figure 1). The analysis methods employed were consistent for both groups of discs to assess the propensity for pellicle formation on the surface following decontamination. Discs with no treatment (pellicle formation) were negative controls, while discs without pellicles were positive controls.

### 2.3. X-ray Photoelectron Spectroscopy (XPS)—Analysis of Chemical Surface Composition

The XPS analysis was conducted on an Axis UltraDLD XP spectrometer (Kratos Analytical Limited, Manchester, UK). The emission of the photoelectrons from the sample was 90° (normal to the sample surface), and the incidence angle of the X-rays was 33.3° (or 56.7° between the X-ray incidence direction and captured photoelectron emission direction). A hybrid lens mode was used with a slot aperture (analysis area of 700 × 300 µm^2^). Survey spectra were acquired with 80 eV pass energy between 0 eV and 1100 eV binding energy (BE), and detail spectra were recorded for O 1s, C 1s, Ti 2p, and N 1s with 40 eV pass energy. The instrument resolution was 1.1 eV for the survey scans and 0.71 eV for the detail scans for the employed settings, determined by measuring the full width at half maximum FWHM of the Ag 3d_5/2_ peak obtained on sputter-cleaned silver foil.

The samples were mounted on an insulating support, and low energy electrons were applied for charge compensation; this combination mitigates differential charging effects due to potential insulating surface layers. Charge referencing of the spectra was based on the position of the C 1s peak attributed to aliphatic C–C/C–H bonds, set to 284.8 eV BE.

### 2.4. Cell Culture and Cytotoxicity Assay

ISO 10993-5:2009 outlines the test procedures for evaluating the in vitro cytotoxicity of medical devices. It involves incubating cultured cells with a device or extracts from a device by direct contact or diffusion. In this study, a murine calvaria-derived preosteoblasts cell line, MC3T3-E1 (ATCC, Manassas, VA, USA), was used to investigate the cytotoxicity of the gels. Cells were cultured in MEM alpha (A10490-01; Gibco, Waltham, MA, USA) supplemented with 15% fetal bovine serum (10270-106, Gibco), 100 U/mL penicillin and 100 µg/mL streptomycin (15140-122; Gibco) in a humidified atmosphere of 5% CO_2_ at 37 °C. Cells were seeded at a density of 3 × 10^4^ cells/cm^2^ in culture-treated plates and allowed to settle for 48 h before treatment. To investigate the cytotoxic effects of the gels, the 1 mL gels were allowed to solidify for 10 min before an equal amount of culture medium (1 mL) was added for 24 h. The conditioned media was added to the cells. After 24 h, 50 μL media was collected, and 2% Triton X-100 (2840; J. T. Baker, Phillipsburg, NJ, USA) was added to the cells for one h to ensure optimal LDH release. Cells not exposed to Triton X-100 had the media exchanged with a new conditioned medium for an additional 24 h, after which 50 μL media was collected, and cells exposed to Triton X-100 for one h. Cells exposed to a culture medium were used as a negative control. Following the manufacturer’s description, cytotoxicity was evaluated using a lactate dehydrogenase (LDH) activity kit (11644793001; Roche, Basel, Switzerland). In short, 50 μL of the collected media was mixed with 50 μL of the reaction mixture and incubated at room temperature in the dark for 30 min. The absorbance was measured at 490 nm using BioTek ELx800 Absorbance Microplate Reader (BioTek Instruments, Inc., Winooski, VT, USA). LDH activity was measured after 24 h and 48 h. The LDH activity was compared in each well before and after Triton X-100 to evaluate the normal LDH release compared to optimal release from dead cells.

### 2.5. Clinical Safety Assessment

Pre-operative: Twelve patients were recruited for the safety assessment study with an open non-comparative case series design. The Regional ethics committee approved the study, REK Sør-Øst (348696), The Norwegian Medicines Agency, NOMA (21/23242-27), and the Norwegian Centre for Research Data (NSD) approved the data handling. The ethics committee did not permit a comparison of different decontamination gels until NuBone^®^ Clean demonstrated no adverse reactions. Consequently, no comparison was made among the different products.

Patients were included if they met the following criteria:

- Good general health
- Between 18 and 65 years of age
- Healthy oral tissues.
- At least ten remaining teeth and/or fixed implants.
- No active oral pathologies.
- Signed Informed Consent was obtained before the start.
- Psychological appropriateness.
- Consent to complete follow-up interview.

Patients were excluded if they met any of the following conditions:

- Not optimal general health condition
- Abscess or infection anywhere in the body at the time of study entry.
- Current pregnancy or nursing.

Patients were excluded if they met any of the following conditions:

- Not optimal general health condition
- Abscess or infection anywhere in the body at the time of study entry.
- Current pregnancy or nursing.
- Any condition or current treatment for any condition, which in the opinion of the investigator and/or consulting physician, may constitute an unwarranted risk.
- The presence of psychological characteristics such as inappropriate attitude or motivation, which, in the investigator’s opinion, are incompatible with the risks involved with the cleaning procedure and the prosthesis.
- Unwillingness to undergo a post-procedure interview.
- If, in the medical opinion of the dental professional, conditions are such that dental cleaning is deemed unsuitable for the patient.
- Intake of Non-Steroidal Anti-Inflammatory Drugs (NSAID), pain-killers or antibiotics one week before and three days after the procedure.

Two investigators conducted the safety study: one principal investigator and one clinical investigator. Preoperatively, the principal investigator provided the study candidate with comprehensive information regarding the research and presented the informed consent form, which occurred at least one week before the initiation of the study. In addition, the clinical investigator reviewed the signed patient information form with the patient during the pre-operative consultation to ensure that the patient understood the content and complied with the instructions.

The Pre-operative Patient History Record (CRF-1) recorded routine clinical examinations and information. The patient and study numbers were given on all Case Report Forms. An identification log was kept at the clinic. The gel in the syringe and the liquid in the vial were mixed according to the instruction. The activated cleaning gel was then applied onto the surfaces of teeth and implants to be cleaned. The gel was applied for 1 min during cleaning before removal by suction accompanied with air and water spray. Patient satisfaction and function were scored in a designated form during the postoperative follow-up interviews. Clinical assessment was done immediately after the procedure and two days after the procedure.

### 2.6. Statistics

Data are presented as mean with standard deviation. The groups were statistically compared using the One-Way and Two-Way ANOVA analysis parametrical test with Tukey’s multiple comparison test. A probability of less than or equal to 0.05 was considered significant. All data obtained were analysed using GraphPad Prism version 9.5 (GraphPad Software Inc., San Diego, CA, USA). All graphical representations were performed on GraphPad Prism and Biorender.

## 3. Results

### 3.1. XPS

XPS provided a detailed surface chemistry analysis on the atomic level measurement of the element and its chemical state after pellicle removal (Table 2) and re-establishment of pellicle on the dental implant surface (Table 3). Table 4 presents the quantification of surface elements for the controls.

After decontamination, the carbon content was highest for PrefGel^®^ (61.9 ± 1.9) and Perisolv^®^ (62.2 ± 8.8). Individually, H_2_O_2_ and Pluronic^®^ had distinct impacts on carbon levels, resulting in values of 50.0 ± 2.8 and 56.5 ± 4.0, respectively. Their combination exhibited a synergistic effect, as demonstrated by the results obtained with NuBone^®^Clean (38.8 ± 1.9), giving the lowest carbon content (Table 2). After re-contamination with saliva (Table 3), H_2_O_2_ (47.6 ± 3.3) and NuBone^®^Clean (48.2 ± 2.2) had the lowest carbon content. Perisolv^®^ had a lower carbon content after re-contamination (49.7 ± 4.5) than after decontamination (Table 2 and Table 3).

The lowest nitrogen level was found for NuBone^®^Clean (2.2 ± 0.1) after decontamination. After re-contamination, none of the groups had significantly lower nitrogen content than the uncleaned control (Table 3).

The highest amount of oxygen was found on NuBone^®^Clean, both after decontamination (43.4 ± 1.0) and re-contamination (33.7 ± 1.9). Perisolv^®^ did show a higher value after re-contamination (33.6 ± 3.7) than decontamination (28.6 ± 2.2).

Traces of silicon were found after decontamination. Other trace elements such as phosphorous, sulphur, chlorine and potassium were found in some of the groups (Table 2 and Table 3). Calcium was found elevated for NuBone^®^Clean and H_2_O_2_. PrefGel had the lowest calcium levels (Table 2 and Table 3). Calcium was elevated for Perisolv^®^ after re-contamination (Table 3).

Titanium content was highest for NuBone^®^Clean, both after decontamination (7.9 ± 2.2) and after re-contamination (3.6 ± 0.9). Perisolv^®^ did show a higher titanium content after re-contamination (2.3 ± 1.8) than after decontamination (3.0 ± 0.4). Table 2 and Table 3.

**Figure 2:**
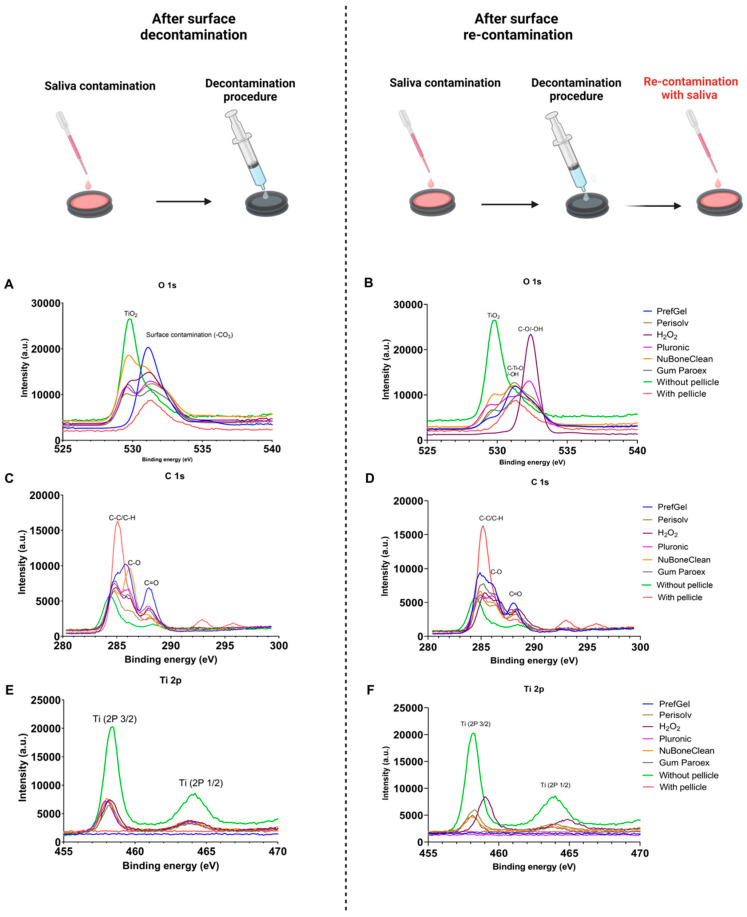
XPS high-resolution spectra of Oxygen (O 1s) (**A**,**B**), Carbon (C 1s) (**C**,**D**), and Titanium (Ti 2p) (**E**,**F**). The left panel are titanium surface after pellicle decontamination, and the right panel are titanium surface after re-contamination with pellicle.

Figure 2 shows XPS high-resolution spectra of Oxygen (O 1s) (Figure 2A,B), Carbon (C 1s) (Figure 2C,D), and Titanium (Ti 2p) (Figure 2E,F), where the left panel are titanium surfaces after pellicle decontamination, and the right panel are titanium surfaces after re-contamination.

After decontamination, the clean control and NuBone^®^Clean exhibited prominent peaks at 529.7 eV BE (Figure 2A). This peak corresponds to O^2−^ in the TiO_2_ [17,18]. The other groups exhibited this peak to a smaller extent. The peak at 531.1 eV BE was prominent for PrefGel^®^ after decontamination and can be attributed to carbon oxides (-CO_3_) [19], which is visible for the different groups to a smaller extent.

After re-contamination, the peak at 529.7 was lower for NuBone^®^Clean than after decontamination. And still prominent for clean control. The peak representing carbon dioxides was lower in the re-contamination for PrefGel^®^ and like the other groups (Figure 2B). A peak at 532.4 was prominent for H_2_O_2_ after re-contamination, representing hydroxyl [17] or surface active oxygen in O-C groups [20]. Peaks at 531.2 for multiple groups (GumParoex^®^, NuBone^®^Clean and PrefGel^®^) can be attributed to C-Ti-O [21] or –OH [22].

After decontamination (Figure 2C), 284.7 to 285.2 peaks representing C–C and C–H bonds [21] were seen in all groups except the clean surface. The clean surface peaked at 284.3, attributed to the C–C bond. It can be hard to differentiate between C–H and C–C bonds [21]. A peak at 286.2 prominent in the NuBone^®^Clean group can be attributed to C-O [21]. A peak of 288 was prominent in PrefGel^®^ and visible in all groups except for positive control, attributed to C=O [21].

After re-contamination, peaks of 284.7 to 285.2 represents C–C and C–H bonds, consistent with the findings during decontamination. A shoulder of 286.2 is more prominent for PrefGel^®^ after re-contamination. A 288 (C=O) peak is still prominent in PrefGel^®^ after re-contamination—Figure 2D. The controls without a pellicle exhibited the most distinct titanium 2P^3/2^ and 2P^½^ peaks, measured at Binding Energies (B.E) of 458.4 and 464.1, respectively [23]. The intensity of these peaks decreased with the presence of a pellicle (Figure 2E,F). In re-contamination, we also see a peak at 459.1 for H_2_O_2_, attributed to TiO_2_; some authors attribute this to Ti-SI bonds [24].

**Figure 3:**
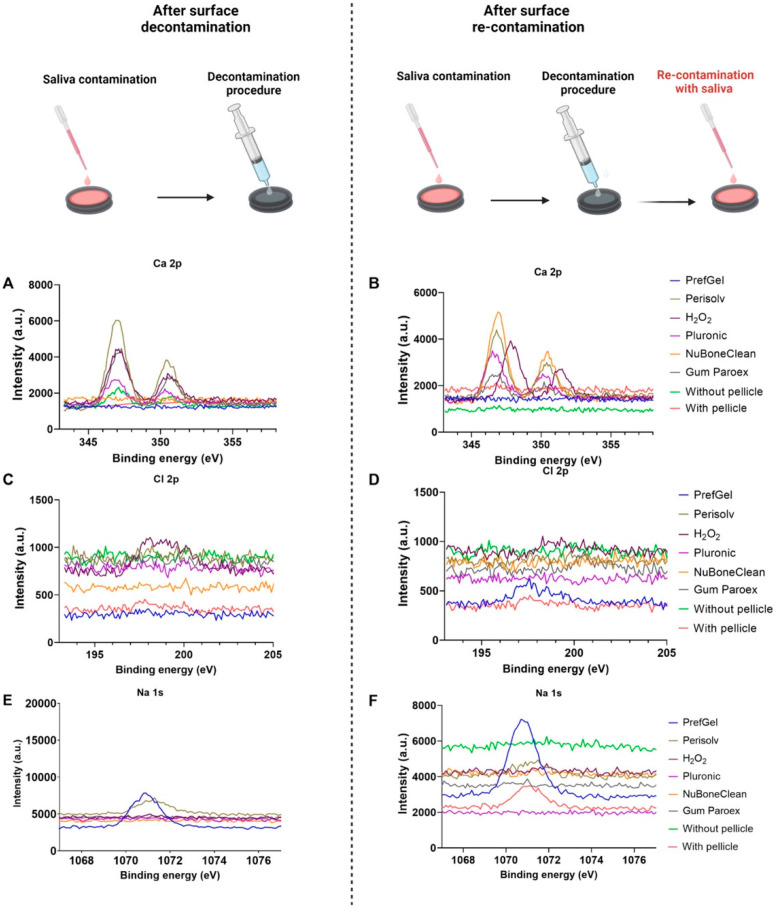
XPS high-resolution spectra of calcium (Ca 1s) (**A**,**B**), chlorine (C1s) (**C**,**D**), and sodium (Na 1s) (**E**,**F**). The left panel are titanium surface after pellicle decontamination, and the right panel are titanium surface after re-contamination with pellicle.

Figure 3 shows the XPS high-resolution spectra of calcium (Ca 2p) (Figure 3A,B), chlorine (Cl 2p) (Figure 3C,D), and sodium (Na 1s) (Figure 3E,F). Again, the left panel are titanium surface after pellicle decontamination, and the right panel are titanium surface after re-contamination with pellicle. Some calcium peaks were visible for Perisolv^®^, H_2_O_2_, Pluronic^®^, GumParoex^®^ and NuBone^®^Clean, whereas no Ca signal was detected from PrefGel^®^ and the controls (Figure 3A,B). The chlorine content was too low to yield clear peaks (Figure 3C,D). Some sodium level was found for PrefGel^®^ in both steps and for Perisolv^®^ after decontamination (Figure 3E,F).

**Figure 4:**
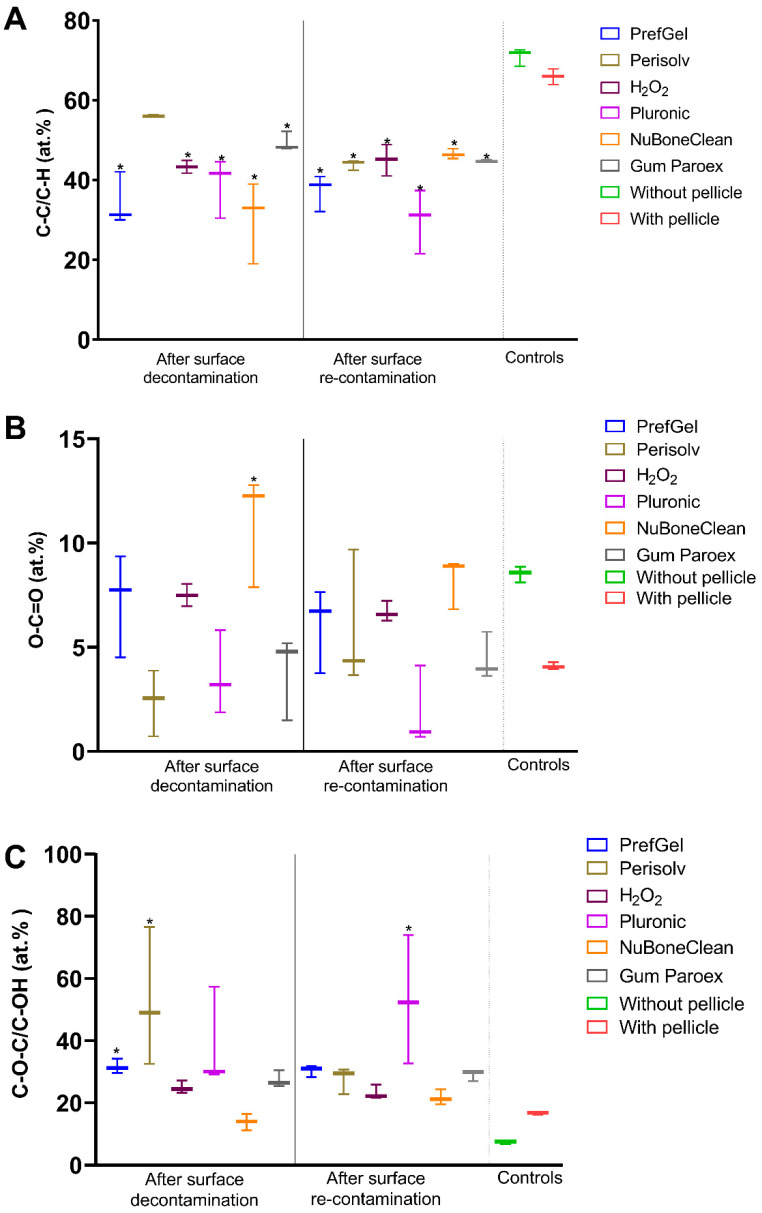
Quantification of selected carbon chemical bonds, C–C/C–H (**A**), O-C=O (**B**) and C-O-C/C-OH (**C**), from the XPS high-resolution spectra (n = 3), * *p* < 0.05 versus titanium surface with a pellicle.

Figure 4 shows the quantification of C–C/C–H, O-C=O and C-O-C/C-OH, calculated from all samples of each group. For C–C/C–H state of carbon shown in Figure 4A, the highest amount was found for Perisolv^®^ after decontamination (56 ± 0.23). All the other groups are significantly lower than the negative control after decontamination and re-contamination. However, both controls have a high atomic%.

For O-C=O, NuBone^®^Clean was statistically significantly different from the negative control (Figure 4B) after decontamination.

C-O-C/C-OH elements were closest for NuBone^®^Clean to the clean control; however, the negative control also has a low value, similar to the positive control. Perisolv^®^, PrefGel^®^ showed high values of these bonds. After re-contamination Pluronic^®^ was significantly higher than the unclean control (Figure 4C).

**Figure 5:**
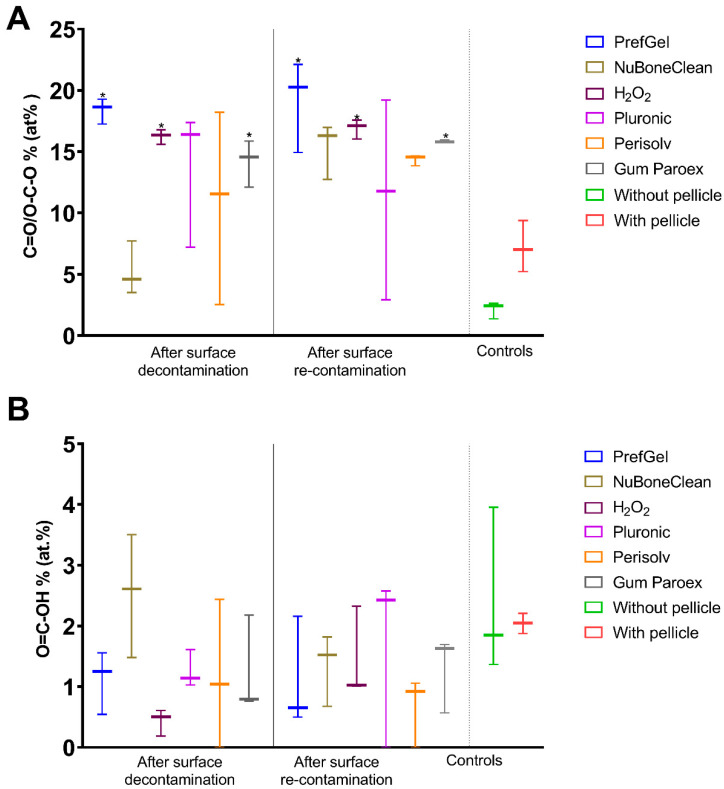
Quantification of selected carbon chemical bonds, C=O/O-C-O (**A**) and O=C-OH (**B**)**,** from the XPS high-resolution spectra (n = 3), * *p* < 0.05 versus titanium surface with a pellicle.

Figure 5 shows the C=O/O-C-O and O=C-OH bonds. No significant difference was found for O=C-OH bonds (Figure 5B); for the C=O/O-C-O, differences were detected for PrefGel^®^, H_2_O_2_ and GumParoex^®^ from the unclean surface. However, all mean values were above negative controls and far from positive ones. Perisolv^®^ had a low value after decontamination.

### 3.2. Cytotoxicity

**Figure 6:**
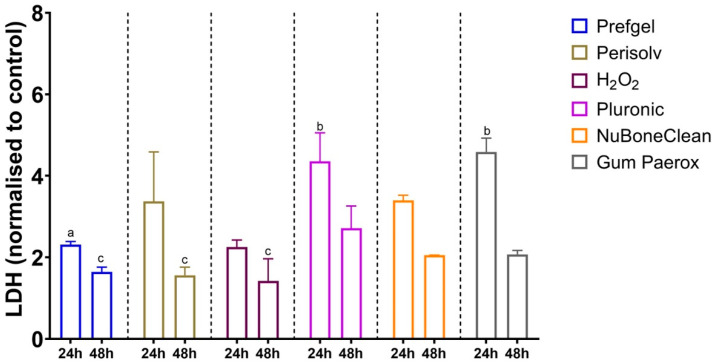
LDH after 24 and 48 h after exposure of decontamination solution to pre-osteoblastic cells, a: significantly lower than GumParoex^®^ at 24 h, b: significantly higher than H_2_O_2_ at 24 h, c: significantly lower than Pluronic^®^ at 48 h.

None of the tested chemical decontamination agents showed cytotoxic behaviour according to ISO 10993-5:2009 (Figure 6); however, the cytotoxic response was highest for Perisolv^®^, Pluronic^®^ and GumPaerox^®^ after 24 h. H_2_O_2_ and PrefGel^®^ had the lowest LDH value. All LDH values decreased from 24 to 48 h of exposure.

### 3.3. Clinical Safety Assessment

**Figure 7:**
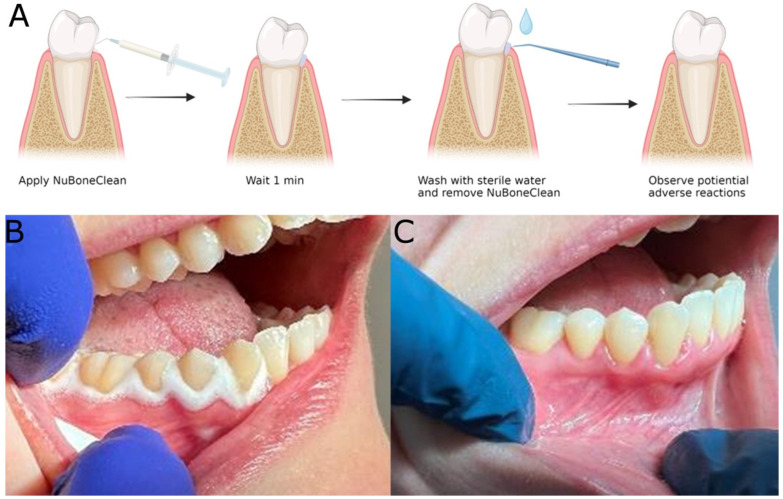
Clinical experiments for detection of potential adverse reactions. (**A**) Graphical presentation of the clinical experiment. (**B**) After application of NuBone^®^ Clean Advanced, (**C**) Clinical control after two days.

All 12 patients included complied with the study protocol. No complications were observed during the clinical procedure, and none of the patients reported any symptoms or discomfort during the study period (Figure 7). NuBone^®^Clean foams, when applied (Figure 7B), were easily removed with water and air spray in combination with suction. The gingiva had normal colour upon control (Figure 7C) for all patients. All 12 patients scored “No” on the presence of gingival (1) redness, (2) swelling, (3) infection, (4) ulceration, (5) ischemia, as well as (6) local allergic reaction, (7) systemic allergic reaction, (8) pain, (9) use of painkillers and (10) use of antibiotics at the day two control.

All patients reported being satisfied with the clinical procedure and had no complaints about the product. Eleven out of 12 patients gave a score of 10. One patient gave a score of 5. The mean score was 9.6, whereas the median score was 10 (Table 5).

## 4. Discussion

Titanium has become the material of choice for bone implants [15]. Although titanium implants have demonstrated impressive success rates in osseointegration [25,26], biofilm-induced infections remain a significant concern in implantology, especially for dental implants. Unlike orthopaedic implants, dental implants are exposed to the oral cavity, which harbours a diverse population of microorganisms capable of adhering to the implant surface. The problem arises when biofilm develops on the implant surface, which interacts with the host immune system and ultimately results in bone loss and implant failure [27]. Therefore, when cleaning a dental implant surface, the goal is to achieve a clean surface that does not attract bacterial cells while simultaneously promoting the attachment of cells crucial for peri-implant tissue regeneration [7].

The initial layer, the acquired pellicle, is crucial for biofilm formation. After cleaning implant components above the bone level, an acquired pellicle is rapidly formed on the implant surface in the oral cavity [11]. The surface chemistry of the implants significantly influences the interaction between titanium implants and the surrounding biological environment [28]. Therefore, it is crucial to evaluate the surface when assessing chemical decontamination products for treating peri-implant diseases or preserving peri-implant health. Among the products tested, Perisolv^®^ and PrefGel^®^ exhibited the lowest levels of titanium measured on the surface, while NuBone^®^Clean had the highest measured content. A higher titanium content indicates a less contaminated surface, as it is closer to the surface without a pellicle (Table 4). In addition, the titanium content and carbon content showed an inverse relationship among the products (Table 2 and Table 3). This implies that the products with the highest measured titanium content, closer to the clean surface, benefit the environment surrounding the implant. In the decontamination phase, the highest content was attributed to NuBone^®^Clean, while in the re-contamination phase, H_2_O_2_ alone has the highest content; however, both values are close to each other. Interestingly Perisolv^®^ had a higher content after re-contamination than after decontamination. It indicated that this product’s effect is visible first when re-contaminating the surface. 

NuBone^®^Clean also exhibited the lowest content of nitrogen on the surface after decontamination. Nitrogen is a common component in proteins [29], and the low nitrogen content observed indicates the effectiveness of surface cleaning. Interestingly, Perisolv^®^ exhibited a lower nitrogen content after re-contamination than after decontamination, further highlighting the compound’s impact after surface re-contamination. This suggests that Perisolv^®^ remains on the surface, exerting a prolonged effect. Similarly, the calcium content on the surface followed a similar pattern, with the highest levels observed for NuBone^®^Clean. After re-contamination, Perisolv^®^ showed higher calcium content than after decontamination, although still lower than NuBone^®^Clean. Calcium is beneficial as numerous calcium-binding proteins play essential roles in cellular processes [30]. Some authors have also indicated that calcium bonding to the implant surface can facilitate bone generation around implants [31]. These findings suggest that NuBone^®^Clean effectively reduces nitrogen content and exhibits higher calcium content on the implant surface, having potential clinical benefits concerning surface cleaning and bone regenerating effects. 

These findings have significant implications when considering early treatment of peri-implant diseases. In previous studies, Pluronic^®^, a polymeric hydrogel, has shown favourable effects on wound healing [32,33]. It belongs to the class of triblock copolymers, which consists of poly(ethylene oxide) (PEO) and poly(propylene oxide) (PPO) blocks arranged in a PEO-PPO-PEO structure. This helps emulsify and solubilise organic contaminants as surfactants, facilitating their removal from the titanium surface. Its use as a wound cleanser in chronic wounds with delayed healing has demonstrated positive outcomes [33]. One of the active ingredients in NuBone^®^Clean, hydrogen peroxide, an oxidising agent, can react with organic contaminants and the Pluronic^®^ hydrogel, forming new functional groups such as the O-C=O bond. The O-C=O group may be formed through the oxidation of hydroxyl groups (C-OH) in PEO and PPO blocks or from the oxidation of organic contaminants on the titanium surface. NuBone^®^Clean showed high values of this bond, close to the clean control. In addition, NuBone^®^Clean exhibited the lowest content of C-OH and C-O-C bonds, again being closest to the clean control.

The presence of specific functional groups, such as C=O and O-C-O can affect the implant’s performance and the biological response it elicits. Following exposure to saliva protein, the emergence of these carbon compounds on titanium surfaces is likely due to the adsorption and subsequent chemical reaction of these proteins on the titanium surface [34]. Saliva proteins are rich in amino acids, many containing carbonyl and ether groups. When these proteins encounter the titanium surface, they may undergo adsorption processes, enriching the surface with these functional groups. This can change the surface chemistry and thus influence how the implant interacts with the surrounding tissues, potentially affecting its performance and biocompatibility, potentially leading to improved re-osseointegration.

Furthermore, saliva proteins may also undergo conformational changes upon interaction with the titanium surface, exposing additional functional groups initially hidden in the protein structure [35]. NuBone^®^Clean had the lowest overall median of C=O and O-C-O measured, being closer to a clean implant surface, indicating less contaminants on the surface. On the other hand, O-C-O groups might improve the resistance to corrosion due to their stability, providing a protective effect on the implant surface. Such conformational changes could contribute to the increase in the presence of C=O and O-C-O groups on the surface for some products.

Overall, the findings elucidate the advantageous effects of combining Pluronic^®^ gel with H_2_O_2_ on pellicle structures. A safety assessment is necessary despite the potential clinical benefits of this combined effect on the pellicle. Patient safety stands dominant in the execution of dental procedures and treatments. As healthcare providers, our commitment lies in ensuring the effectiveness of our materials and strategies while preserving patient well-being. None of the tested decontamination solutions were cytotoxic.

Moreover, the clinical assessment of safety did not exhibit any adverse reactions. However, it is incumbent upon us to proceed with caution and to pursue further research before incorporating these solutions into clinical practice confidently. Studying the proteins that adhere to the implant surface remains necessary post-treatment. This investigation will explore whether these proteins encourage regeneration or biofilm formation. In furthering our understanding, controlled randomised clinical trials should be undertaken to evaluate this product’s clinical efficacy, especially in combination with mechanical treatment modalities.

Developing new remedies for peri-implant disease treatment is urgent due to the increasing prevalence of dental implants and the subsequent rise in implant-related complications [36]. A recent case study utilising electrolytic cleaning revealed the potential for bone regeneration in patients with peri-implantitis [37]. Nevertheless, the absence of a control group underscores the need for additional evaluation in this field. As dental practitioners, we are responsible for providing our patients with the most effective and safe treatments. However, the quest for more effective treatments should never compromise safety. We observed in this study that products like PrefGel^®^, typically used on root surfaces and applied to implant surfaces, did not have any beneficial effects on the implant surface—mainly underlining the necessity for more comprehensive testing of dental biomaterials. Conclusively, our discussion underscores the necessity for larger, more extensive studies to substantiate our findings and solidify the foundation for clinical recommendations. Although our research is an encouraging pilot study in evaluating these chemical agents’ safety and potential clinical benefits, we recognise the imperative of validating our findings through more comprehensive clinical trials.

## 5. Conclusions

In conclusion, the evaluation of surface chemistry can contribute to a better understanding of the effect of chemical decontamination products for titanium implants. Our study underscores the potential of chemical decontamination solutions in treating peri-implant disease. We have presented a potential chemical decontamination methodology that is effective against pellicle formation and is safe with limitations to the in vitro and human testing protocols. NuBone^®^Clean was more effective in providing a cleaner titanium surface than other tested chemical decontamination candidates. None of the tested chemical decontamination candidates had any cytotoxic effect. Furthermore, we advocate for larger studies to evaluate the safety and efficacy of these chemical agents thoroughly before they are introduced to the market. This multifaceted approach will ensure that the development and deployment of new dental treatments are safe and effective, thereby protecting patient well-being while advancing the field of dentistry.

## Figures and Tables

**Table 1:** Decontamination products were used in this study.

Product Name	Content	In Clinical Dental Use
PrefGel^®^	24% EDTA + hydrogel	Yes
Perisolv^®^	Sodium hypochlorite + a hydrogel	Yes
Hydrogen peroxide	3% H_2_O_2_ in water	Yes
Pluronic^®^ F-127	28% Poloxamer in water	No
NuBone^®^ Clean	3% H_2_O_2_ + a hydrogel (poloxamer)	No
GUM^®^ Paroex^®^	0.12% Chlorhexidine digluconate + 0.05% Cetylpyridinium chloride	Yes

**Table 2:** Element quantification of titanium surfaces after pellicle removal with the decontamination products (n = 3), * *p* < 0.05 versus an untreated titanium surface (i.e., surface with pellicle).

** 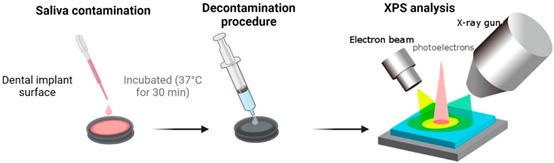 **						
**Elemental Analysis after Decontamination (Atomic% ± Standard Deviation)**						
**Element**	**PrefGel**	**Perisolv**	**H_2_O_2_**	**Pluronic**	**NuBoneClean**	**GumPareox**
C 1s%	61.9 ± 1.9	62.2 ± 8.8	50.0 ± 2.8 *	56.5 ± 4.0 *	38.8 ± 1.9 *	56.0 ± 1.7 *
N 1s%	10.0 ± 0.7	5.1 ± 3.7	8.5 ± 0.5	7.2 ± 2.1	2.2 ± 0.1 *	8.7 ± 0.0
O 1s%	26.1 ± 1.6 *	28.6 ± 2.2 *	32.3 ± 1.8 *	30.0 ± 1.0 *	43.4 ± 1.0 *	28.1 ± 1.4 *
Na 1s%	0.9 ± 1.3	0.0 ± 0.0	0.0 ± 0.0	0.0 ± 0.0	0.0 ± 0.0	0.0 ± 0.0
Si 2p%	0.9 ± 0.5	0.1 ± 0.1	0.5 ± 0.2	0.4 ± 0.3	0.7 ± 0.0	0.1 ± 0.1
P 2p%	0.0 ± 0.0	0.5 ± 0.4	1.0 ± 0.3	0.4 ± 0.2	1.7 ± 0.4	0.5 ± 0.1
S 2p%	0.0 ± 0.0	0.0 ± 0.0	0.2 ± 0.1	0.1 ± 0.0	0.0 ± 0.0	0.1 ± 0.1
Cl 2p%	0.0 ± 0.0	0.6 ± 0.0	0.4 ± 0.3	0.0 ± 0.0	0.0 ± 0.0	0.1 ± 0.0
K 2p%	0.0 ± 0.0	0.0 ± 0.0	0.1 ± 0.1	0.0 ± 0.0	0.0 ± 0.0	0.0 ± 0.0
Ca 2p%	0.0 ± 0.0	1.2 ± 0.9	2.6 ± 0.5 *	1.2 ± 0.1	4.6 ± 0.7 *	1.0 ± 0.1
Ti 2p%	0.0 ± 0.1	2.3 ± 1.8	4.6 ± 1.4 *	4.1 ± 1.4 *	7.9 ± 2.2 *	5.3 ± 0.6 *

**Table 3:** Element quantification of titanium surfaces after re-exposure to pellicle after applying decontamination products (n = 3), * *p* < 0.05 versus titanium surface untreated titanium surface (i.e., surface with pellicle).

** 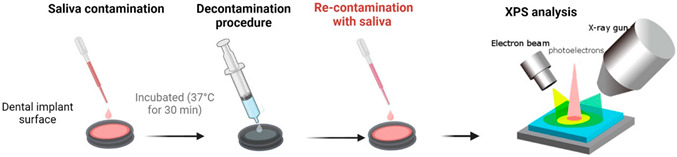 **						
**Elemental Analysis after Both Decontamination and Re-Contaminations with Saliva** **(Atomic% ± Standard Deviation)**						
**Element**	**PrefGel**	**Perisolv**	**H_2_O_2_**	**Pluronic**	**NuBoneClean**	**GumPareox**
C 1s%	63.4 ± 2.2	49.7 ± 4.5 *	47.6 ± 3.3 *	60.8 ± 9.2	48.2 ± 2.2 *	57.7 ± 2.1 *
N 1s%	10.8 ± 0.4	7.4 ± 1.1	8.6 ± 1.3	4.9 ± 3.7	8.4 ± 0.5	10.2 ± 0.2
O 1s%	24.6 ± 1.4 *	33.6 ± 3.7 *	33.4 ± 2.3 *	29.7 ± 3.1 *	33.7 ± 1.9 *	26.4 ± 1.2 *
Na 1s%	0.0 ± 0.0	0.0 ± 0.0	0.0 ± 0.0	0.0 ± 0.0	0.0 ± 0.0	0.0 ± 0.0
Si 2p%	0.1 ± 0.2	0.4 ± 0.2	0.2 ± 0.2	0.2 ± 0.2	0.1 ± 0.2	0.0 ± 0.0
P 2p%	0.1 ± 0.1	1.3 ± 0.2 *	1.6 ± 0.7	0.6 ± 0.4	1.7 ± 0.4	0.7 ± 0.2
S 2p%	0.0 ± 0.0	0.0 ± 0.0	0.2 ± 0.1	0.0 ± 0.0	0.1 ± 0.1	0.1 ± 0.1
Cl 2p%	0.2 ± 0.1	0.3 ± 0.3	0.4 ± 0.2	0.0 ± 0.0	0.0 ± 0.0	0.1 ± 0.0
K 2p%	0.1 ± 0.1	0.4 ± 0.2	0.6 ± 0.6	0.0 ± 0.0	0.0 ± 0.0	0.0 ± 0.0
Ca 2p%	0.1 ± 0.1	3.0 ± 0.4 *	2.8 ± 0.9 *	1.2 ± 0.6	3.6 ± 0.9 *	1.7 ± 0.4
Ti 2p%	0.5 ± 0.6	3.8 ± 2.0 *	4.7 ± 0.3 *	2.5 ± 1.3	4.1 ± 1.4 *	3.0 ± 0.7

**Table 4:** Quantification of surface elements for the controls, titanium surfaces with and without pellicle (n = 3).

	Controls (at% ± SD)	
Element	Without Pellicle	With Pellicle
C 1s%	32.1 ± 0.5	73.4 ± 3.3
N 1s%	0.9 ± 0.1	6.1 ± 0.8
O 1s%	47.6 ± 0.5	17.1 ± 1.8
Na 1s%	0.0 ± 0.0	0.0 ± 0.0
Si 2p%	0.2 ± 0.2	0.0 ± 0.0
P 2p%	0.2 ± 0.2	0.4 ± 0.1
S 2p%	0.0 ± 0.0	0.4 ± 0.1
Cl 2p%	0.1 ± 0.2	0.1 ± 0.0
K 2p%	0.0 ± 0.1	2.2 ± 0.6
Ca 2p%	0.0 ± 0.1	0.1 ± 0.1
Ti 2p%	18.7 ± 0.3	0.0 ± 0.0

**Table 5:** Patient satisfaction Score.

Patient	Patient Satisfaction Score
1	10
2	10
3	10
4	10
5	10
6	10
7	10
8	10
9	10
10	5
11	10
12	10
Mean	9.6
Median	10.0

## Data Availability

Data is available upon request to the corresponding author.
